# Glucose Intolerance and Cancer Risk: A Community-Based Prospective Cohort Study in Shanghai, China

**DOI:** 10.3389/fonc.2021.726672

**Published:** 2021-08-30

**Authors:** Juzhong Ke, Tao Lin, Xiaolin Liu, Kang Wu, Xiaonan Ruan, Yibo Ding, Wenbin Liu, Hua Qiu, Xiaojie Tan, Xiaonan Wang, Xi Chen, Zhitao Li, Guangwen Cao

**Affiliations:** ^1^Department of Epidemiology, Second Military Medical University, Shanghai, China; ^2^Pudong New Area Center for Disease Control and Prevention, Pudong Institute of Preventive Medicine, Fudan University, Shanghai, China

**Keywords:** type 2 diabetes mellitus, prediabetes, cancer, prospective cohort study, cancer prevention

## Abstract

**Background:**

Cancer becomes the leading cause of premature death in China. Primary objective of this study was to determine the major risk factors especially glucose intolerance for cancer prophylaxis.

**Methods:**

A cluster sampling method was applied to enroll 10,657 community-based adults aged 15-92 years in Shanghai, China in 2013. A structured questionnaire and physical examination were applied in baseline survey. Prediabetes was diagnosed using 75-g oral glucose tolerance test. After excluding 1433 subjects including 224 diagnosed with cancer before and 1 year after baseline survey, the remaining 9,224 subjects were followed-up to December 31, 2020.

**Results:**

A total of 502 new cancer cases were diagnosed. The cancer incidence was 10.29, 9.20, and 5.95/1,000 person-years in diabetes patients, those with prediabetes, and healthy participants, respectively (*p*<0.001). The multivariate Cox regression analysis indicated that age, prediabetes and diabetes, were associated with an increased risk of cancer in those <65 years, the hazard ratios (95% confidence interval) for prediabetes and diabetes were, 1.49(1.09-2.02) and 1.51(1.12-2.02), respectively. Glucose intolerance (prediabetes and diabetes) were associated with increased risks of stomach cancer, colorectal cancer, and kidney cancer in those <65 years. Anti-diabetic medications reduced the risk of cancer caused by diabetes. The multivariate Cox analysis showed that age, male, <9 years of education, and current smoking were associated with increased risks of cancer in those ≥65 years independently.

**Conclusions:**

Glucose intolerance is the prominent cancer risk factor in adults <65 years. Lifestyle intervention and medications to treat glucose intolerance help prevent cancer in this population.

## Introduction

With the socioeconomic development, cancer has become the first leading cause of premature death (death before the mean life of a given population) in the most regions of China including Shanghai (1). The occurrence profiles of all cancer and site-specific cancers are changing, especially in younger adults. Incidence among this population is increasing for some site-specific cancers related to metabolic syndrome but decreasing for some cancers associated with infections or smoking (2–4). Update of controllable risk factor exposure is extremely important for the specific prophylaxis of cancer in population with an altered socioeconomic situation.

Type 2 diabetes mellitus and cancer are the major health problems worldwide. The age-standardized incidence of diabetes keeps increasing (5). Given that a substantial number of cancer cases are attributable to diabetes in different populations (6, 7), the increase in diabetes-related health burden and its impact on cancer risk represents an ongoing challenge. However, studies that examined cancer risk before diabetes diagnosis are relatively rare. Prediabetes is an often undiagnosed condition lasts for an average duration of 9.5 years before clinical onset of diabetes (8). Some reported indicated that prediabetes may increase the overall cancer risk (9, 10). However, many studies have failed to determine the role of prediabetes and diabetes on the risk of cancer (11–13). Thus, more reliable prospective cohort studies are needed to consolidate the etiological relationship between cancer and glucose intolerance, especially at a pre-diabetic level. Furthermore, C-reactive protein (CRP), a general marker of chronic low-grade inflammation, is associated with multiple chronic diseases including diabetes (14). CRP might have a joint effect with metabolic syndrome in carcinogenesis (15). It remains to determine if CRP contributes to carcinogenesis independently. Long-term use of metformin, an anti-diabetic, has been associated with a decreased risk of cancer, possibly because metformin works directly to cancer cells and/or the microenvironment (16–18). More recently, sulfonylureas, another groups of anti-diabetics, has been demonstrated to increase the risk of colorectal cancer in diabetes patients (19). Thus, the association of anti-diabetic medications with cancer risk remains controversial.

In this community-based prospective cohort study, we aimed to identify holistic risk factors especially glucose intolerance that can be applied for active prophylaxis of cancer in young adults and elderly adults, respectively. The study subjects aged between 15 years and 64 year were defined as young adults, while those aged 65 years or older were defined as elderly adults, according to the previous reports (20, 21). This study is of significance for cancer prophylaxis in the modern society, especially for the prevention of cancer-related premature death.

## Materials and Methods

### Participants

This community-based prospective cohort study was performed in Pudong New Area, Shanghai, China. Participants are permanent residents who possess Shanghai household registration. Multistage stratified random cluster sampling was employed to sample study participants. A total of 38 urban streets and rural townships in Pudong were stratified into 3 strata according to the socioeconomic disparities from the Yearbook of Pudong government. Four streets in each stratum (6 urban streets and 6 rural townships) were randomly selected. Second, 16 urban communities and 18 rural villages were randomly selected from the 6 urban streets and 6 rural towns, respectively. Third, 11.0% families in each community/village were randomly selected. Individuals with diagnosed type I diabetes and pregnant women were excluded from this survey. A total of 12,382 eligible adults aged between 15 years and 92 years were initially recruited, among whom 10657 agreed to participate the study.

### Baseline Survey

Baseline survey was carried out between January 13^th^ and July 30^st^, 2013. Demographic characteristics including age, sex, marital status, years of education, lifestyle factors including smoking, alcohol consumption, tea consumption, physical activity, and preexisting medical conditions including family history of cancer, history of viral hepatitis, chronic atrophic gastritis, and use of anti-inflammatory agents were collected using a structured questionnaire (**Supplementary Table 1**). This face-to-face interview was conducted by trained investigators working in the community health centers. Current smoking was defined as smoking at least one cigarette a day in the past 6 months. Alcohol consumption and tea consumption were defined as regular drinker with at least three times per week in the past 6 months. Physical activity was defined as participating in sports activity for at least once per week in the past 5 years. Cancer family history was defined as at least one first-degree relative diagnosed with cancer.

All participants were invited to take physical examinations. Glucose, lipids, and CRP in the fasting plasma were measured using a HITACHI 7170A automatic biochemical analyzer. Glucose metabolism was determined using a 75g-oral glucose tolerance test (OGTT). Diabetes was defined as fasting plasma glucose ≥7.0 mmol/L, a 2-h plasma glucose ≥11.1 mmol/L by OGTT test, or on a glucose control medication. Participants with fasting plasma glucose between 6.1 mmol/L and 7.0 mmol/L and 2h plasma glucose <7.8 mmol/L were diagnosed as impaired fasting glucose (IFG). Participants with fasting plasma glucose <6.1mmol/L and 2h plasma glucose between 7.8 mmol/L and 11.1 mmol/L were diagnosed as impaired glucose tolerance (IGT). Both IFG and IGT are categorized as prediabetes (22). Participants with fasting plasma glucose <6.1 mmol/L and 2h plasma glucose <7.8 mmol/L were categorized as normal glucose tolerance (NGT). Body mass index (BMI) was calculated as weight (kg)/height (m^2^). Hypertension was defined as blood pressure ≥140/90mm Hg or on a blood pressure-lowering medication. Dyslipidemia was defined as participants with plasma triglyceride ≥2.26mmol/L, total cholesterol ≥6.20mmol/L, low-density lipoprotein (LDL) ≥4.13mmol/L, high-density lipoprotein (HDL) <1.03mmol/L or on a cholesterol-lowering medication.

### Follow-Up

The participants were excluded if confirmed not to possess Shanghai household registration (n=233), not to complete questionnaire and physical examination (n=976), and to have diagnosed cancer previously (n=170). The participants were also excluded if being diagnosed with cancer within the first year of follow-up (n=54). The remaining 9,224 eligible subjects (3,395 men and 5,829 women) were followed-up every three years. The flow diagram is shown in **Supplementary Figure 1**. Information on time-varying, physician-diagnosed incident diabetes, use of anti-diabetic medications, and covariates was obtained using a questionnaire during follow-up. The study protocol conformed to the 1975 Declaration of Helsinki and was approved by the ethics committee of the Center for Disease Control and Prevention of the Pudong New Area, Shanghai, China. A signed informed consent was obtained from each participant.

The outcomes of this cohort study are the incidences of all-cause primary cancers. Incident cancer cases were annually verified by data linkage with the cancer registration and management system in Shanghai, China. This system has covered 100% of registered population since 2002. The data in this system are reliable and their quality has been approved by the World Health Organization (23). Site-specific cancer types were identified according to the International Classification of Diseases, 10th edition (ICD-10), as previously described (1).

### Statistical Analysis

For each participant, the expected number of person-years of follow-up for cancer incidence was calculated as the total years between their exact age at baseline survey and their exact age at cancer diagnosis, death, or 31st December 2020, whichever came first. Patients died of conditions unrelated to cancer were censored. One-way ANOVA test and Kruskal-Wallis test were applied to compare continuous variables. Difference in categorical variables was determined using chi-square test. Hazard ratio (HR) and 95% confidence intervals (CI) were calculated using the Cox proportional hazard model. Study participants were stratified into young adults and elderly adults. Baseline glycemic status, together with other variables including age, sex, marriage status, years of education, BMI, current smoking, alcohol consumption, tea consumption, physical activity, family history of cancer, history of hypertension, dyslipidemia, viral hepatitis, chronic atrophic gastritis, use anti-inflammatory agents, and serum CRP were introduced into the Cox proportional hazard model. The significant factors in the univariate Cox regression analysis were introduced into the multivariate Cox model to determine the factors independently associated with cancer. The Kaplan-Meier method was applied to estimate the effect of the factor proven to be significant in the Cox regression analysis on the cumulative incidence of cancer. Interaction terms were added in models to test the potential interactions of these covariates with baseline glycemic status. SPSS version 22.0 (SPSS Inc., Chicago, IL) was applied for statistical analysis. All statistical tests were two-sided. A *p* value of <0.05 was considered to be statistically significant.

## Results

### Baseline Characteristics

Age and sex distribution of study subjects are shown in **Supplementary Figure 2**. In this cohort, 1454 participants (15.76%) were diagnosed with prediabetes, 1790 participants (19.41%) were diagnosed with diabetes at baseline. Baseline characteristics of the participants stratified by glycemic status are presented in **Table 1**. Compared to the NGT participants, those with prediabetes or diabetes were older and had higher frequencies of hypertension and dyslipidemia and higher levels of triglycerides, total cholesterol, LDL, CRP, and BMI and a lower level of HDL. Physical activity, history of viral hepatitis, and family history of cancer did not differ between the NGT participants and those with glucose intolerance (prediabetes + diabetes) statistically.

**Table 1:** Baseline participant characteristics stratified by glycemic status.

Age group (Years old)	Characteristics	Glycemic status			Total	*p*
		NGT	Prediabetes	Diabetes		
15-64	Age (years)	50.81 ± 11.18	55.29 ± 7.63	56.05 ± 6.77	52.25 ± 10.41	<0.001^$ 1*** ^3^***^
	Male (%)	1538 (32.72%)	309 (33.70%)	440 (42.19%)	2287 (34.33%)	<0.001^§ 1* ^2^*^
	Urban (%)	2818 (59.94%)	496 (54.09%)	583 (55.90%)	3897 (58.50%)	0.001^§ 1* ^3^*^
	Married (%)	4215 (89.66%)	867 (94.55%)	976 (93.58%)	6058 (90.95%)	<0.001^§ 1* ^3^*^
	>= 9 years of education (%)	4186 (89.04%)	762 (83.10%)	858 (82.26%)	5806 (87.16%)	<0.001^§ 1* ^3^*^
	Current smoking (%)	817 (17.38%)	146 (15.92%)	255 (24.45%)	1218 (18.29%)	<0.001^§ 1* ^2^*^
	Alcohol consumption (%)	524 (11.15%)	126 (13.74%)	155 (14.86%)	805 (12.09%)	0.001^§ 3*^
	Tea consumption (%)	1320 (28.08%)	265 (28.90%)	361 (34.61%)	1946 (29.21%)	<0.001^§ 1* ^2^*^
	Physical activity (%)	1194 (25.40%)	210 (22.90%)	258 (24.74%)	1662 (24.95%)	0.274^§^
	Family history of cancer (%)	286 (6.08%)	77 (8.40%)	58 (5.56%)	421 (6.32%)	0.017^§ 1* ^2^*^
	Hypertension (%)	1245 (26.48%)	443 (48.31%)	565 (54.17%)	2253 (33.82%)	<0.001^§ 1* ^2^* ^3^*^
	Dyslipidemia (%)	1901 (40.44%)	529 (57.69%)	650 (62.32%)	3080 (46.24%)	<0.001^§ 1* ^3^*^
	Triglycerides (mmol/L)	1.53 ± 1.29	1.98 ± 1.42	2.25 ± 2.16	1.70 ± 1.51	<0.001^$ 1*** ^2^*** ^3^***^
	Total cholesterol (mmol/L)	5.42 ± 1.10	5.69 ± 1.08	5.71 ± 1.22	5.50 ± 1.12	<0.001^$ 1*** ^3^***^
	LDL (mmol/L)	3.00 ± 0.98	3.33 ± 0.99	3.32 ± 1.03	3.10 ± 1.00	<0.001^$ 1*** ^3^***^
	HDL (mmol/L)	1.41 ± 0.34	1.33 ± 0.34	1.29 ± 0.32	1.38 ± 0.34	<0.001^$ 1*** ^2^** ^3^***^
	BMI (kg/m^2^)	24.39 ± 3.84	25.94 ± 3.57	26.23 ± 3.77	24.89 ± 3.87	<0.001^$ 1*** ^3^***^
	HbA1c (%)	5.18 ± 0.64	5.59 ± 0.81	6.89 ± 1.75	5.51 ± 1.11	<0.001^$ 1*** ^2^*** ^3^***^
	History of viral hepatitis (%)	227 (4.83%)	46 (5.02%)	54 (5.18%)	327 (4.91%)	0.883^§^
	Chronic atrophic gastritis (%)	165 (3.51%)	38 (4.14%)	22 (2.11%)	225 (3.38%)	0.030^§ 2*^
	Use anti-inflammatory agents (%)	113 (2.40%)	40 (4.36%)	62 (5.94%)	215 (3.23%)	<0.001^§ 1* ^3^*^
	CRP (mg/L)	0.95 ± 3.56	1.59 ± 5.14	1.84 ± 4.55	1.17 ± 3.99	<0.001^# 1*** ^2^*** ^3^***^
≥ 65	Age (years)	71.58 ± 5.92	72.68 ± 6.20	72.50 ± 5.98	72.08 ± 6.01	<0.001^$ 1** ^3^**^
	Male (%)	569 (44.49%)	235 (43.76%)	304 (40.70%)	1108 (43.23%)	0.242^§^
	Urban (%)	829 (64.82%)	341 (63.50%)	478 (63.99%)	1648 (64.30%)	0.848^§^
	Married (%)	1034 (80.84%)	410 (76.35%)	575 (76.97%)	2019 (78.77%)	0.037^§^
	≥9 years of education (%)	780 (60.99%)	292 (54.38%)	393 (52.61%)	1465 (57.16%)	<0.001^§ 1* ^3^*^
	Current smoking (%)	159 (12.43%)	77 (14.34%)	90 (12.05%)	326 (12.72%)	0.434^§^
	Alcohol consumption (%)	165 (12.90%)	74 (13.78%)	76 (10.17%)	315 (12.29%)	0.098^§^
	Tea consumption (%)	313 (24.47%)	133 (24.77%)	190 (25.44%)	636 (24.81%)	0.889^§^
	Physical activity (%)	397 (31.04%)	135 (25.14%)	183 (24.50%)	715 (27.90%)	0.002^§ 1* ^3^*^
	Family history of cancer (%)	76 (5.94%)	23 (4.28%)	37 (4.95%)	136 (5.31%)	0.311^§^
	Hypertension (%)	652 (50.98%)	332 (61.82%)	540 (72.29%)	1524 (59.46%)	<0.001^§ 1* ^2^* ^3^*^
	Dyslipidemia (%)	603 (47.15%)	297 (55.31%)	446 (59.71%)	1346 (52.52%)	<0.001^§ 1* ^3^*^
	Triglycerides (mmol/L)	1.47 ± 0.81	1.75 ± 1.17	1.91 ± 1.35	1.66 ± 1.09	<0.001^$ 1*** ^2^* ^3^***^
	Total cholesterol (mmol/L)	5.59 ± 1.11	5.64 ± 1.12	5.70 ± 1.12	5.63 ± 1.11	0.112^$^
	LDL (mmol/L)	3.15 ± 1.02	3.20 ± 1.03	3.26 ± 1.04	3.19 ± 1.03	0.058^$^
	HDL (mmol/L)	1.39 ± 0.34	1.33 ± 0.32	1.31 ± 0.31	1.35 ± 0.33	<0.001^$ 1** ^3^***^
	BMI (kg/m^2^)	24.87 ± 3.29	25.41 ± 3.72	26.00 ± 3.49	25.31 ± 3.47	<0.001^$ 1** ^2^** ^3^***^
	HbA1c (%)	5.33 ± 0.66	5.65 ± 0.80	6.75 ± 1.53	5.81 ± 1.19	<0.001^$ 1*** ^2^*** ^3^***^
	History of viral hepatitis infection (%)	45 (3.52%)	15 (2.79%)	25 (3.35%)	85 (3.32%)	0.732^§^
	Chronic atrophic gastritis (%)	68 (5.32%)	23 (4.28%)	22 (2.95%)	113 (4.41%)	0.042^§ 3*^
	Use anti-inflammatory agents (%)	87 (6.80%)	38 (7.08%)	67 (8.97%)	192 (7.49%)	0.186^§^
	CRP (mg/L)	1.31 ± 3.95	1.82 ± 5.79	2.15 ± 5.71	1.66 ± 4.94	0.001^# 1*** ^3^***^
Total	Age (years)	55.25 ± 13.35	61.71 ± 11.02	62.92 ± 10.37	57.76 ± 12.93	<0.001^$ 1*** ^2^* ^3^***^
	Male (%)	2107 (35.23%)	544 (37.41%)	744 (41.56%)	3395 (36.81%)	<0.001^§ 2* ^3^*^
	Urban (%)	3647 (60.99%)	837 (57.57%)	1061 (59.27%)	5545 (60.11%)	0.041^§^
	Married (%)	5249 (87.78%)	1277 (87.83%)	1551 (86.65%)	8077 (87.57%)	0.424^§^
	≥9 years of education (%)	4966 (83.04%)	1054 (72.49%)	1251 (69.89%)	7271 (78.83%)	<0.001^§ 1* ^3^*^
	Current smoking (%)	976 (16.32%)	223 (15.34%)	345 (19.27%)	1544 (16.74%)	0.004^§ 2* ^3^*^
	Alcohol consumption (%)	689 (11.52%)	200 (13.76%)	231 (12.91%)	1120 (12.14%)	0.035^§^
	Tea consumption (%)	1633 (27.31%)	398 (27.37%)	551 (30.78%)	2582 (27.99%)	0.014^§ 3*^
	Physical activity (%)	1591 (26.61%)	345 (23.73%)	441 (24.64%)	2377 (25.77%)	0.038^§^
	Family history of cancer (%)	362 (6.05%)	100 (6.88%)	95 (5.31%)	557 (6.04%)	0.174^§^
	Hypertension (%)	1897 (31.72%)	775 (53.30%)	1105 (61.73%)	3777 (40.95%)	<0.001^§ 1* ^2^* ^3^*^
	Dyslipidemia (%)	2504 (41.87%)	826 (56.81%)	1096 (61.23%)	4426 (47.98%)	<0.001^§ 1* ^2^* ^3^*^
	Triglycerides (mmol/L)	1.52 ± 1.21	1.90 ± 1.34	2.11 ± 1.87	1.69 ± 1.40	<0.001^$ 1*** ^2^*** ^3^***^
	Total cholesterol (mmol/L)	5.45 ± 1.10	5.67 ± 1.09	5.70 ± 1.18	5.54 ± 1.12	<0.001^$ 1*** ^3^***^
	LDL (mmol/L)	3.03 ± 0.99	3.28 ± 1.01	3.30 ± 1.04	3.12 ± 1.01	<0.001^$ 1*** ^3^***^
	HDL (mmol/L)	1.40 ± 0.34	1.33 ± 0.33	1.29 ± 0.31	1.37 ± 0.34	<0.001^$ 1*** ^2^** ^3^***^
	BMI (kg/m^2^)	24.49 ± 3.73	25.75 ± 3.63	26.14 ± 3.66	25.01 ± 3.77	<0.001^$ 1*** ^2^** ^3^***^
	HbA1c (%)	5.22 ± 0.65	5.61 ± 0.80	6.83 ± 1.66	5.59 ± 1.14	<0.001^$ 1*** ^2^*** ^3^***^
	History of viral hepatitis infection (%)	272 (4.55%)	61 (4.20%)	79 (4.41%)	412 (4.47%)	0.837^§^
	Chronic atrophic gastritis (%)	233 (3.90%)	61 (4.20%)	44 (2.46%)	338 (3.66%)	0.009^§ 2* ^3^*^
	Use anti-inflammatory agents (%)	200 (3.34%)	78 (5.36%)	129 (7.21%)	407 (4.41%)	<0.001^§ 1* ^3^*^
	CRP (mg/L)	1.02 ± 3.65	1.67 ± 5.39	1.97 ± 5.07	1.31 ± 4.28	<0.001^# 1*** ^2^*** ^3^***^

# Comparison performed using Kruskal-Wallis test. § Comparison performed using Chi-square test. $ Comparison performed using one-way ANOVA test.

Data are n (%) or mean ± SD.

P value indicates the statistical result for the Kruskal-Wallis, chi-square or one-way ANOVA test. The results of Post hoc multiple comparisons (Bonferroni) were indicated as follows: 1, NGT versus prediabetes; 2, prediabetes versus diabetes; 3, NGT versus diabetes. *P < 0.05; **P < 0.01; ***P < 0.001.

NGT, normal glucose tolerance; LDL, low-density lipoprotein; HDL, high-density lipoprotein; HbA1c, glycated hemoglobin A_1c_; BMI, body mass index; CRP, C-reactive protein.

### Association Between Glycemic Status and Cancer Incidence

Over a median of 7.48 years follow-up, cancer was found in 502 participants. The cumulative incidence of total cancer per 1,000 person-years in the participants with diabetes, those with prediabetes, and those with NGT was 10.29, 9.20, and 5.95 (log-rank test *p* value <0.001). In the multivariate Cox regression analysis, the interaction of age and glycemic status was significantly associated with an increased risk of cancer (*p*_interaction_ = 0.040). The associations of all the variables with cancer risk were initially evaluated in the univariate Cox regression analysis. It was found that age, prediabetes, diabetes, BMI, hypertension, and CRP were significantly associated with an increased risk of total cancer in young adults. The multivariate Cox regression analysis demonstrated that age, prediabetes and diabetes independently associated with an increased risk of total cancer after the adjustment for the above significant variables in this population. In elderly adults, age, male, <9 years of education, and current smoking were independently associated with an increased risk of total cancer in the multivariate Cox regression analysis. Age, diabetes and current smoking were independently associated with an increased risk of all cancer in all the study population (**Table 2**).

**Table 2:** Cox regression analysis of factors significantly affected cancer incidence in cohort participants, stratified by age group.

Age at the baseline	Variable	Persons at risk	Incident casess	Person-years	Incidence (1/1000)	Univariate analysis		Multivariate Analysis*	
						HR (95% CI)	*p*	HR (95% CI)	*p*
15-64 years	Glycemic status								
	NGT	4701	169	35880	4.71	ref.		ref.	
	Prediabetes	917	57	6892	8.27	1.76 (1.30-2.37)	<0.001	1.49 (1.09-2.02)	0.012
	Diabetes	1043	67	7808	8.58	1.82 (1.37-2.42)	<0.001	1.51 (1.12-2.02)	0.006
	Age								
	15-24	155	2	1203	1.66	ref.		ref.	
	25-34	416	4	3223	1.24	0.74 (0.14-4.06)	0.733	0.73 (0.13-3.99)	0.717
	35-44	741	11	5731	1.92	1.15 (0.26-5.20)	0.853	1.09 (0.24-4.94)	0.908
	45-54	1746	66	13303	4.96	2.98 (0.73-12.15)	0.129	2.69 (0.66-11.04)	0.168
	55-64	3603	210	27120	7.74	4.65 (1.16-18.73)	0.030	4.13 (1.02-16.75)	0.047
	Sex								
	Male	2287	97	17351	5.59	ref.		–	–
	Female	4374	196	33230	5.90	1.05 (0.83-1.34)	0.672	–	–
	Area								
	Urban	3897	170	29686	5.73	ref.		–	–
	Rural	2764	123	20895	5.89	0.98 (0.78-1.23)	0.850	–	–
	Marriage status								
	Married	6058	270	45987	5.87	ref.		–	–
	Other	603	23	4594	5.01	0.85 (0.56-1.30)	0.462	–	–
	Years of education								
	≥9	5806	251	44094	5.69	ref.		–	–
	<9	855	42	6486	6.48	1.14 (0.82-1.58)	0.434	–	–
	BMI	6661	293	50581	5.79	1.03 (1.00-1.06)	0.033	1.02 (0.98-1.05)	0.310
	Current smoking								
	No	5443	232	41383	5.61	ref.		–	–
	Yes	1218	61	9198	6.63	1.18 (0.89-1.57)	0.241	–	–
	Alcohol consumption								
	No	5856	252	44482	5.67	ref.		–	–
	Yes	805	41	6098	6.72	1.19 (0.85-1.65)	0.308	–	–
	Tea consumption								
	No	4715	198	35846	5.52	ref.		–	–
	Yes	1946	95	14734	6.45	1.17 (0.91-1.49)	0.214	–	–
	Physical activity								
	No	4999	224	37936	5.90	ref.		–	–
	Yes	1662	69	12644	5.46	0.92 (0.71-1.21)	0.569	–	–
	Family history of cancer								
	No	6264	270	47574	5.68	ref.		–	–
	Yes	397	23	3006	7.65	1.34 (0.88-2.06)	0.172	–	–
	Hypertension								
	No	4408	177	33575	5.27	ref.		ref.	
	Yes	2253	116	17006	6.82	1.29 (1.02-1.63)	0.031	0.90 (0.70-1.15)	0.393
	Dyslipidemia								
	No	3581	143	27257	5.25	ref.		–	–
	Yes	3080	150	23324	6.43	1.23 (0.98-1.54)	0.081	–	–
	Viral hepatitis								
	No	6334	275	48119	5.72	ref.		–	–
	Yes	327	18	2462	7.31	1.28 (0.79-2.06)	0.313	–	–
	Chronic atrophic gastritis								
	No	6436	282	48884	5.77	ref.		–	–
	Yes	225	11	1697	6.48	1.12 (0.62-2.05)	0.704	–	–
	HbA1c	6661	293	50581	5.79	1.09 (0.99-1.19)	0.068	–	–
	Use anti-inflammatory agents								
	No	6446	282	48966	5.76	ref.		–	–
	Yes	215	11	1615	6.81	1.18 (0.65-2.16)	0.584	–	–
	CRP	6661	293	50581	5.79	1.02 (1.00-1.04)	0.014	1.02 (1.00-1.03)	0.072
≥65 years	Glycemic status								
	NGT	1279	100	9303	10.75	ref.		–	–
	Prediabetes	537	42	3864	10.87	1.01 (0.70-1.45)	0.954	–	–
	Diabetes	747	67	5218	12.84	1.20 (0.88-1.63)	0.257	–	–
	Age								
	65-74	1704	114	12565	9.07	ref.		ref.	
	75-84	775	85	5332	15.94	1.76 (1.33-2.33)	<0.001	1.60 (1.18-2.16)	0.002
	≥85	84	10	488	20.48	2.28 (1.20-4.36)	0.012	1.94 (0.99-3.79)	0.054
	Sex								
	Male	1108	106	7796	13.60	ref.		ref.	
	Female	1455	103	10589	9.73	0.71 (0.54-0.94)	0.015	0.71 (0.51-1.00)	0.048
	Area								
	Urban	1648	125	11855	10.54	ref.		–	–
	Rural	915	84	6531	12.86	0.82 (0.62-1.08)	0.158	–	–
	Marriage status								
	Married	2019	155	14684	10.56	ref.		ref.	
	Other	544	44	3702	11.89	1.39 (1.02-1.89)	0.039	1.26 (0.89-1.78)	0.184
	Years of education								
	≥ 9	1465	101	10676	9.46	ref.		ref.	
	< 9	1098	108	7709	14.01	1.48 (1.13-1.95)	0.004	1.44 (1.06-1.95)	0.020
	BMI	2563	209	18386	11.37	1.03 (0.99-1.07)	0.197	–	–
	Current smoking								
	No	2237	164	16103	10.18	ref.		ref.	
	Yes	326	45	2282	19.72	1.94 (1.39-2.69)	<0.001	1.88 (1.29-2.73)	0.001
	Alcohol consumption								
	No	2248	175	16171	10.82	ref.		–	–
	Yes	315	34	2214	15.36	1.42 (0.98-2.05)	0.062	–	–
	Tea consumption								
	No	1927	164	13794	11.89	ref.		–	–
	Yes	636	45	4591	9.80	0.82 (0.59-1.14)	0.247	–	–
	Physical activity								
	No	1848	151	13179	11.46	ref.		–	–
	Yes	715	58	5206	11.14	0.97 (0.72-1.32)	0.856	–	–
	Family history of cancer								
	No	2433	200	17430	11.47	ref.		–	–
	Yes	130	9	956	9.42	0.82 (0.42-1.59)	0.552	–	–
	Hypertension								
	No	1039	83	7490	11.08	ref.		–	–
	Yes	1524	126	10896	11.56	1.04 (0.79-1.38)	0.763	–	–
	Dyslipidemia								
	No	1217	109	8659	12.59	ref.		–	–
	Yes	1346	100	9727	10.28	0.82 (0.62-1.07)	0.140	–	–
	Viral hepatitis								
	No	2478	199	17790	11.19	ref.		–	–
	Yes	85	10	596	16.79	1.50 (0.80-2.83)	0.209	–	–
	Chronic atrophic gastritis								
	No	2450	198	17572	11.27	ref.		–	–
	Yes	113	11	814	13.52	1.20 (0.65-2.20)	0.559	–	–
	HbA1c	2563	209	18386	11.37	1.01 (0.90-1.13)	0.882	–	–
	Use anti-inflammatory agents								
	No	2371	194	17033	11.39	ref.		–	–
	Yes	192	15	1353	11.09	0.97 (0.58-1.65)	0.920	–	–
	CRP	2563	209	18386	11.37	1.00 (0.98-1.03)	0.807	–	–
Total	Glycemic status								
	NGT	5980	269	45184	5.95	ref.		ref.	
	Prediabetes	1454	99	10756	9.20	1.55 (1.23-1.95)	<0.001	1.24 (0.98-1.58)	0.072
	Diabetes	1790	134	13027	10.29	1.73 (1.41-2.13)	<0.001	1.42 (1.10-1.82)	0.006
	Age								
	15-24	155	2	1203	1.66	ref.		ref.	
	25-34	416	4	3223	1.24	0.75 (0.14-4.07)	0.734	0.77 (0.14-4.26)	0.766
	35-44	741	11	5731	1.92	1.15 (0.26-5.20)	0.853	1.18 (0.26-5.42)	0.832
	45-54	1746	66	13303	4.96	2.98 (0.73-12.15)	0.129	2.95 (0.71-12.3)	0.137
	55-64	3603	210	27120	7.74	4.65 (1.16-18.72)	0.030	4.60 (1.12-18.92)	0.035
	65-74	1704	114	12565	9.07	5.46 (1.35-22.09)	0.017	5.18 (1.25-21.48)	0.023
	75-84	775	85	5332	15.94	9.64 (2.37-39.17)	0.002	8.78 (2.11-36.46)	0.003
	≥85	84	10	488	20.48	12.60 (2.76-57.51)	0.001	11.75 (2.53-54.57)	0.002
	Sex								
	Male	3395	203	25147	8.07	ref.		–	–
	Female	5829	299	43819	6.82	0.84 (0.71-1.01)	0.062	–	–
	Area								
	Urban	5545	295	41541	7.10	ref.		–	–
	Rural	3679	207	27426	7.55	0.94 (0.79-1.13)	0.523	–	–
	Marriage status								
	Married	8077	425	60671	7.01	ref.		ref.	
	Other	1147	77	8296	9.28	1.33 (1.04-1.69)	0.022	1.16 (0.89-1.51)	0.283
	Years of education								
	≥9	7271	352	54771	6.43	ref.		ref.	
	<9	1953	150	14196	10.57	1.65 (1.36-2.00)	<0.001	1.11 (0.90-1.38)	0.339
	BMI	9224	502	68966	7.28	1.03 (1.01-1.06)	0.004	1.03 (1.00-1.05)	0.056
	Current smoking								
	No	7680	396	57486	6.89	ref.		ref.	
	Yes	1544	106	11480	9.23	1.34 (1.08-1.66)	0.007	1.44 (1.14-1.83)	0.002
	Alcohol consumption								
	No	8104	427	60654	7.04	ref.		ref.	
	Yes	1120	75	8313	9.02	1.28 (1.00-1.64)	0.047	1.10 (0.84-1.43)	0.493
	Tea consumption								
	No	6642	362	49641	7.29	ref.		–	–
	Yes	2582	140	19326	7.24	0.99 (0.82-1.21)	0.944	–	–
	Physical activity								
	No	6847	375	51116	7.34	ref.		–	–
	Yes	2377	127	17850	7.11	0.97 (0.79-1.19)	0.765	–	–
	Family history of cancer								
	No	8697	470	65004	7.23	ref.		–	–
	Yes	527	32	3962	8.08	1.11 (0.78-1.59)	0.558	–	–
	Hypertension								
	No	5447	260	41065	6.33	ref.		ref.	
	Yes	3777	242	27901	8.67	1.37 (1.15-1.63)	<0.001	0.92 (0.76-1.11)	0.363
	Dyslipidemia								
	No	4798	252	35916	7.02	ref.		–	–
	Yes	4426	250	33050	7.56	1.08 (0.90-1.28)	0.404	–	–
	Viral hepatitis								
	No	8812	474	65908	7.19	ref.		–	–
	Yes	412	28	3058	9.16	1.27 (0.87-1.86)	0.216	–	–
	Chronic atrophic gastritis								
	No	8886	480	66455	7.22	ref.		–	–
	Yes	338	22	2511	8.76	1.21 (0.79-1.86)	0.377	–	–
	HbA1c	9224	502	68966	7.28	1.09 (1.01-1.16)	0.020	0.94 (0.86-1.03)	0.201
	Use anti-inflammatory agents								
	No	8817	476	65999	7.21	ref.		–	–
	Yes	407	26	2967	8.76	1.22 (0.82-1.80)	0.332	–	–
	CRP	9224	502	68966	7.28	1.02 (1.00-1.03)	0.021	1.01 (0.99-1.02)	0.243

*Only included significant covariates in univariate analysis.

NGT, normal glucose tolerance; BMI, body mass index; HbA1c, glycated hemoglobin A_1c_; CRP, C-reactive protein.

### Effect of Abnormal Glycemic Status and Anti-Diabetic Treatment on Cancer Incidence

We stratified participants with abnormal glycemic status into subgroups. Participants with prediabetes were categorized into IFG only, IGT only, and both IFG and IGT. Participants with diabetes were categorized into previously diagnosed diabetes or detected during baseline screening, use of anti-diabetic medications or not, or duration since the first diagnosis of diabetes. The multivariate Cox regression analysis demonstrated that, compared to participants with NGT at baseline, cancer incidence was significantly higher in prediabetes patients with IFG only, in diabetes patients detected during baseline screening rather than in those diagnosed previously, in diabetes patients without anti-diabetic medications rather than in those receiving regular anti-diabetic medications including insulin, euglycemic agents, sulfonylureas, biguanides, thiazolidinediones, α-glycosidase inhibitors, and Chinese traditional anti-diabetic medicine, or in diabetes patients diagnosed within 5 years rather than in those diagnosed longer than 5 years in whole participants. This effect was only evident in young adults rather than in elderly adults (**Table 3**).

**Table 3:** Effects of glucose intolerance on cancer incidence in the study participants.

Age at the baseline	Variable	Persons at risk	Incident casess	Person-years	Incidence (1/1000)	Univariate analysis		Multivariate Analysis*		
						HR (95% CI)	*p*	HR (95% CI)	*p*
15-64 years	NGT	4701	169	35880	4.71	ref.		ref.	
	Category of prediabetes								
	IFG	323	22	2433	9.04	1.92 (1.23-3.00)	0.004	1.67 (1.07-2.61)	0.023
	IGT	436	27	3265	8.27	1.76 (1.17-2.64)	0.007	1.51 (1.00-2.27)	0.048
	IFG+IGT	158	8	1194	6.70	1.42 (0.70-2.89)	0.331	1.22 (0.60-2.48)	0.581
	Category of diabetes								
	Diagnosed previously	573	31	4308	7.20	1.53 (1.04-2.24)	0.030	1.26 (0.85-1.86)	0.248
	Screen detected at the baseline	470	36	3501	10.28	2.18 (1.52-3.13)	<0.001	1.86 (1.29-2.70)	0.001
	Diabetes patients with anti-diabetic medications								
	Yes	473	29	3538	8.20	1.74 (1.18-2.58)	0.006	1.43 (0.95-2.13)	0.083
	No	570	38	4271	8.90	1.89 (1.33-2.69)	<0.001	1.61 (1.12-2.30)	0.010
	Duration since first diagnose of diabetes								
	<5 years	749	52	5603	9.28	1.97 (1.45-2.69)	<0.001	1.68 (1.22-2.31)	0.002
	≥5 years	294	15	2206	6.80	1.45 (0.85-2.45)	0.171	1.16 (0.68-1.98)	0.590
≥65 years	NGT	1279	100	9303	10.75	ref.		ref.	
	Category of prediabetes								
	IFG	141	14	1027	13.64	1.27 (0.72-2.22)	0.406	–	–
	IGT	308	18	2223	8.10	0.75 (0.46-1.24)	0.268	–	–
	IFG+IGT	88	10	614	16.28	1.51 (0.79-2.90)	0.211	–	–
	Category of diabetes								
	Diagnosed previously	459	32	3231	9.90	0.92 (0.62-1.37)	0.689	0.89 (0.60-1.33)	0.577
	Screen detected at the baseline	288	35	1987	17.62	1.64 (1.12-2.41)	0.012	1.52 (1.03-2.24)	0.033
	Diabetes patients with anti-diabetic medications								
	Yes	378	28	2635	10.63	0.99 (0.65-1.50)	0.960	–	–
	No	369	39	2583	15.10	1.41 (0.97-2.04)	0.071	–	–
	Duration since first diagnose of diabetes								
	<5 years	435	41	3079	13.32	1.24 (0.86-1.78)	0.246	–	–
	≥5 years	312	26	2139	12.15	1.13 (0.74-1.74)	0.573	–	–
Total	NGT	5980	269	45184	5.95	ref.		ref.	
	Category of prediabetes								
	IFG	464	36	3460	10.41	1.75 (1.23-2.48)	0.002	1.50 (1.06-2.13)	0.023
	IGT	744	45	5488	8.20	1.38 (1.00-1.89)	0.047	1.04 (0.75-1.43)	0.810
	IFG+IGT	246	18	1809	9.95	1.67 (1.04-2.69)	0.035	1.32 (0.82-2.14)	0.252
	Category of diabetes								
	Diagnosed previously	1032	63	7539	8.36	1.41 (1.07-1.85)	0.015	1.04 (0.79-1.38)	0.779
	Screen detected at the baseline	758	71	5488	12.94	2.18 (1.68-2.83)	<0.001	1.69 (1.29-2.21)	<0.001
	Diabetes patients with anti-diabetic medications								
	Yes	851	57	6173	9.23	1.55 (1.17-2.07)	0.002	1.15 (0.86-1.54)	0.344
	No	939	77	6854	11.23	1.89 (1.47-2.44)	<0.001	1.45 (1.12-1.89)	0.005
	Duration since first diagnosis of diabetes								
	<5 years	1184	93	8681	10.71	1.8 (1.42-2.28)	<0.001	1.41 (1.11-1.80)	0.005
	≥5 years	606	41	4345	9.44	1.59 (1.14-2.21)	0.006	1.12 (0.80-1.57)	0.520

*Only included significant covariates shown in **Table 2** in univariate Cox regression analysis.

NGT, normal glucose tolerance; IFG, impaired fasting glucose; IGT, impaired glucose tolerance.

### Association of the Incidences of Site-Specific Cancers With Baseline Glycemic Status

**Figure 1:**
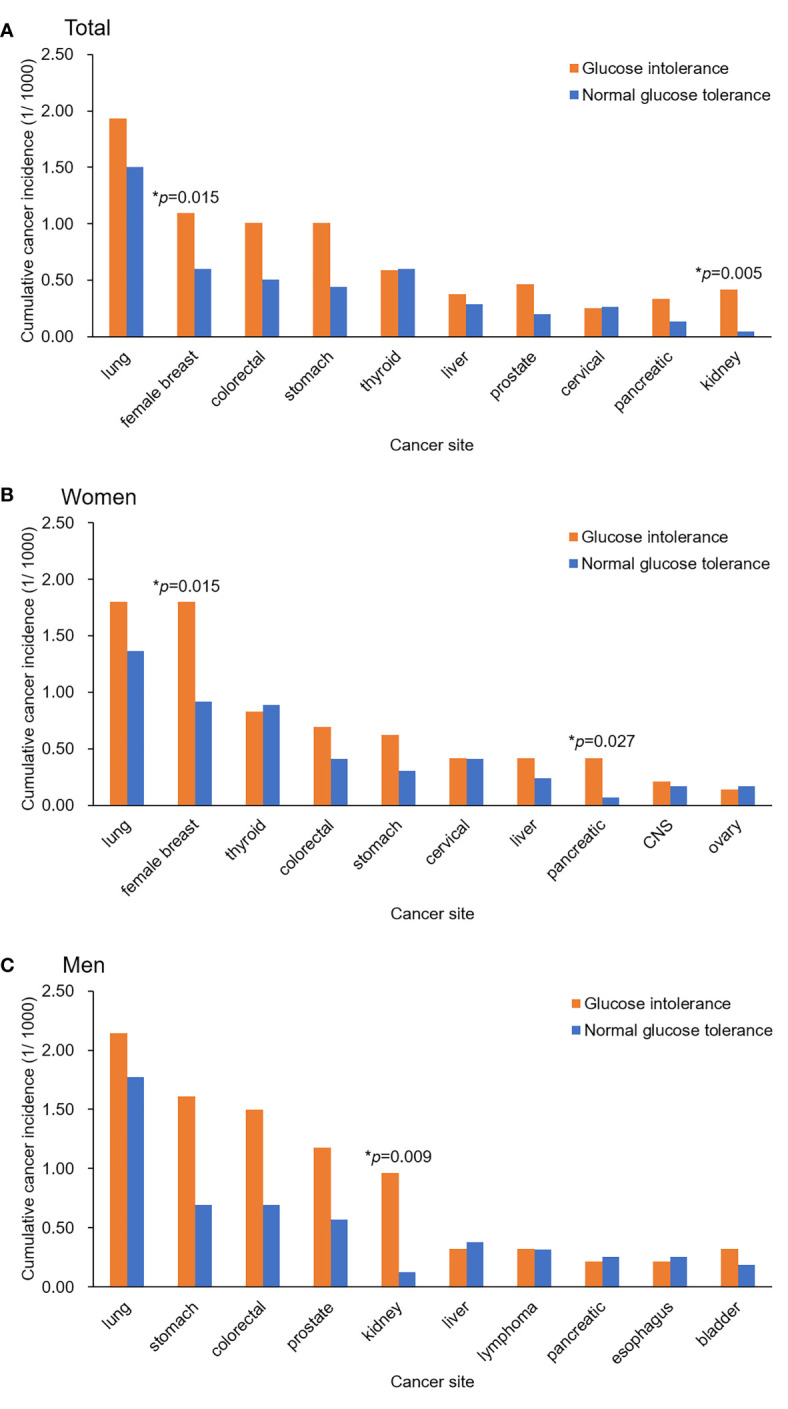
Cumulative incidence rates of the top 10 site-specific cancers during the follow-up among the study participants with different baseline glycemic status. **(A)** Total participants, **(B)** Women, **(C)** Men. Differences in the cumulative incidence rates were tested using a Cox proportional hazards model, adjusted for age and sex.

The association of site-specific cancers with baseline glycemic status in the whole population was first evaluated by the Cox regression analysis, adjusted for age and sex. Female breast cancer, and kidney cancer were significantly associated with glucose intolerance (prediabetes+diabetes) (**Supplementary Table 2** and **Figure 1A**). Women with glucose intolerance had higher incidences of female breast cancer and pancreatic cancer (**Figure 1B**). Men with glucose intolerance had a higher incidence of kidney cancer (**Figure 1C**). Stratification analysis indicated that in the whole population, participants with prediabetes had increased risks of stomach cancer and kidney cancer, while participants with diabetes had increased risks of female breast cancer and kidney cancer (**Supplementary Table 3**). In young adults, glucose intolerance was significantly associated with increased risks of stomach cancer, colorectal cancer, and kidney cancer in the Cox regression analysis, adjusted for age and sex (**Table 4**). Participants with prediabetes had increased risks of stomach cancer, kidney cancer and pancreatic cancer. Participants with diabetes had increased risks of stomach cancer, colorectal cancer and kidney cancer in this population (**Supplementary Table 4**).

**Table 4:** The Cox regression analysis of the association of site-specific cancer with the baseline glycemic status in the adults < 65 years, adjusted for age and sex.

Glycemic status	Persons at risk	Incident cases	Person-years	Incidence (1/1000)	HR (95% CI)	*p*
Lung cancer						
NGT	4701	45	35880	1.25	ref.	
Glucose intolerance	1960	23	14700	1.56	1.04 (0.63-1.73)	0.872
Female breast cancer						
NGT	3163	23	24123	0.95	ref.	
Glucose intolerance	1211	16	9107	1.76	1.44 (0.75-2.75)	0.268
Stomach cancer						
NGT	4701	10	35880	0.28	ref.	
Glucose intolerance	1960	16	14700	1.09	3.72 (1.68-8.20)	0.001
Colorectal cancer						
NGT	4701	9	35880	0.25	ref.	
Glucose intolerance	1960	13	14700	0.88	3.51 (1.50-8.22)	0.004
Kidney cancer						
NGT	4701	2	35880	0.06	ref.	
Glucose intolerance	1960	8	14700	0.54	8.69 (1.84-40.95)	0.006
Liver cancer						
NGT	4701	5	35880	0.14	ref.	
Glucose intolerance	1960	3	14700	0.20	1.47 (0.35-6.14)	0.599
Pancreatic cancer						
NGT	4701	3	35880	0.08	ref.	
Glucose intolerance	1960	4	14700	0.27	3.27 (0.73-14.6)	0.121
Esophageal cancer						
NGT	4701	3	35880	0.08	–	–
Glucose intolerance	1960	0	14700	0.00	–	–

HR, Hazard ratio; NGT, normal glucose tolerance; Glucose intolerance, prediabetes + diabetes.

## Discussion

In this community-based prospective cohort study, diabetes and prediabetes were identified to be independently associated with increased risks of total cancer and site-specific cancers such as stomach cancer, colorectal cancer, and kidney cancer in young adults (<65 years). Anti-diabetic medications reduced the risk of cancer caused by diabetes. The outcomes of this study may reflect the current risk factors of cancer in young adults. The study population was randomly recruited from urban and rural communities in Pudong New Area, the only district with urban and rural residents in Shanghai (21). Pudong New Area has about 5 million permanent residents with diverse socioeconomic status, which is highly representative for other populations. The permanent residents possessing Shanghai household registration were recruited in this study, just because this population could be eligible to be followed-up and information of cancer occurrence could be verified by data linkage with the cancer registration and management system. This does not affect the representativeness. Thus, the findings of this study can be generalized to other populations both within and outside China.

In this study, we demonstrated that glucose intolerance was significantly associated with an increased risk of total cancer especially for stomach cancer, colorectal cancer and kidney cancer in young adults. These effects were independent of other risk factors. In elderly adults, glucose intolerance was not independently associated with increased risk of total cancer. Cancer occurs more often in aged adults than in younger ones. The effect of glucose intolerance on cancer might be covered by the overwhelming effects of age and current smoking in aged adults. Our data support that the risk factors of all cancer have shifted from the pollution and chronic infections in the past decades to metabolic syndrome at the present (23). Metabolic syndrome, which is often caused by overconsumption of calories and fat and lack of physical activity, is prevalent worldwide. An important study has demonstrated that HRs for all-site and site-specific cancers are particularly elevated during the first year following diabetes diagnosis (6). Diabetes is associated with higher risk of colorectal adenomas, a precancerous lesion of colorectal cancer, in adults aged 40-49 years (24). A cross-sectional study using data from the 2001-2014 National Health and Nutrition Examination Survey has shown that individuals <65 years have higher odds of colorectal cancer when also diagnosed with diabetes (25). It has been demonstrated that diabetes patients aged ≤50 or 55 years have a greater risk of all cancers, digestive cancers, and urinary cancers (26, 27). These findings suggest that glucose intolerance may facilitate cancer development in young adults, making this population with glucose intolerance a target population for cancer screening and interventions. Since the incidence of diabetes is increasing dramatically in the younger generation (28, 29), our finding is of public health importance in monitoring all cancer in young adults who have glucose intolerance. Public health actions including encouraging physical activity and restricting energy intake to reduce the prevalent and incident glucose intolerance should be important in reducing cancer risk in young adults.

In this study, we demonstrated that anti-diabetic medications were significantly associated with a decreased risk of all cancer in young adults with diabetes. Interestingly, diabetes patients who were diagnosed previously and diagnosed 5 years or longer did not have an increased risk of all cancer, whereas diabetes patients diagnosed at the baseline survey and within 5 years had an increased risk of cancer (**Table 3**). This is possibly because long-term anti-diabetic medications have been widely applied in the study subjects who were diagnosed as diabetes 5 years ago. Anti-diabetic medications had been covered by basic medical insurance for decades in Shanghai, China. Our result is quite consistent with another cohort study carried out in Italy (30). Lifelong use of anti-diabetics is protective for all cancer in patients with diabetes. We postulate that increase in physical activity and dietary continence should be protective for all cancer in young adults with prediabetes.

The mechanism by which glucose intolerance is associated with an increased risk of all cancer remains largely unknown. Here, we demonstrated that glucose intolerance was associated with increased risks of stomach cancer, colorectal cancer, kidney cancer, and pancreatic cancer in young adults, and female breast cancer, stomach cancer, and kidney cancer in the whole population. Data from the China Kadoorie Biobank Study have shown that glucose intolerance was associated with increased risks of certain site-specific cancers including female breast cancer, liver cancer, pancreatic cancer, and colorectal cancer (6, 31). The findings in Chinese population are mostly consistent with that in Western population (6, 25, 30). The association of glucose intolerance with stomach cancer is not evident in a cohort study in the Northern Swedish population (12), possibly because of the differences in the susceptibility of gastric cancer between study populations. Although each site-specific cancer has its own risk factors, they share a common risk factor: chronic inflammation. Metformin that was proven to inhibit cancer cell growth and modulate cancer microenvironment has been demonstrated to have potent inflammation-inhibitory effects (32). In this study, CRP, a well-established marker of systemic inflammation in metabolic syndrome (33), tend to be identified as an independent risk factor of cancer in young adults. Elevated CRP has been associated with an increased risk of diabetes in middle-aged and elderly Chinese (34). Chronic inflammation related to glucose intolerance might play an essential role in carcinogenesis. Insulin is a potent growth factor that promotes cell proliferation and carcinogenesis directly and/or through insulin-like growth factor 1 (IGF-1). Hyperinsulinemia leads to an increase in the bioactivity of IGF-1 by inhibiting IGF binding protein-1 (35). Apart from directly promotes cancer progression, hyperglycemia increases the levels of insulin/IGF-1 and inflammatory cytokines in circulation (36). Metabolic disorder was associated with increased risk of liver cancer (37). In this study, the association of glucose intolerance with liver cancer was not evident possibly due to few cases of liver cancer diagnosed in this cohort. Even though, glycemic control is important for cancer prevention in young adults.

The strengths of this study include a perspective design, the high representativeness of community-based study population, holistic risk factors screening, use of standard OGTT at the baseline survey, adjustment for multiple potential confounding factors, and reliable follow-up. This study has three main implications. First, young adults with glucose intolerance are recommended to undergo appropriate cancer screenings for early diagnosis. Second, steps to prevent cancer should be taken even at pre-diabetic stage. Some forms of diabetes treatment and a reversal of obesity and prediabetes can reduce cancer risk (38). Glycemic management and lifestyle intervention are of public health significance. Third, this study provides clue to elucidate the mechanism by which glucose intolerance induces carcinogenesis.

### Limitations

This study has several limitations. First, risk factors for cancer were not all included in the baseline survey, such as dietary habit, stress, and social factors, resulting loss of data. Second, the follow-up period was relatively short, resulting in small number of end-point events that weakened the statistical power. Third, information of the income was incomplete because of personal privacy. The education levels might serve as an alternative in this analysis. Fourth, small number of end-point events makes it difficult to investigate the associations of each type of anti-diabetic medicines with the risk of cancer.

## Conclusions

In this community-based prospective cohort study, diabetes and prediabetes were independently associated with increased risks of total cancer and site-specific cancers such as stomach cancer, colorectal cancer, and kidney cancer in young adults. Regular monitoring of plasma glucose level could assist to identify individuals with an increased risk of cancer. Lifestyle interventions and anti-diabetic medications to prevent and treat prediabetes and diabetes are important in cancer prophylaxis in young adults.

## Data Availability Statement

The raw data supporting the conclusions of this article will be made available by the authors, without undue reservation.

## Ethics Statement

The studies involving human participants were reviewed and approved by Ethics committee of the Center for Disease Control and Prevention of the Pudong New Area, Shanghai, China. The patients/participants provided their written informed consent to participate in this study.

## Author Contributions

Conceptualization: JK, TL, XR, and GC. Data curation: JK and GC. Funding acquisition: GC. Investigation: JK, TL, XL, KW, XR, WL, HQ, XT, XW, YD, and GC. Methodology: JK, TL, XL, KW, XR, HQ, XT, XW, XC, and GC. Project administration: TL, XR, and GC. Supervision: GC. Validation: XR, HQ, and XT. Visualization: JK and GC. Writing - original draft and revising: GC.

## Funding

This work was supported by National Natural Scientific Foundation of China grant (grant number: 81520108021, 81673250) to GC.

## Conflict of Interest

The authors declare that the research was conducted in the absence of any commercial or financial relationships that could be construed as a potential conflict of interest.

## Publisher’s Note

All claims expressed in this article are solely those of the authors and do not necessarily represent those of their affiliated organizations, or those of the publisher, the editors and the reviewers. Any product that may be evaluated in this article, or claim that may be made by its manufacturer, is not guaranteed or endorsed by the publisher.

## Supplementary Material

Supplementary Figure 1 Diagram of this community-based prospective cohort study

Supplementary Figure 2 Age and sex distribution of study subjects.

## References

[1] Wang S Du X Han X Yang F Zhao J Li H. Influence of Socioeconomic Events on Cause-Specific Mortality in Urban Shanghai, China, From 1974 to 2015: A Population-Based Longitudinal Study. CMAJ (2018) 190(39):E1153–61. 10.1503/cmaj.180272 PMC6167223 30274992

[2] Heer EV Harper AS Sung H Jemal A Fidler-Benaoudia MM. Emerging Cancer Incidence Trends in Canada: The Growing Burden of Young Adult Cancers. Cancer (2020) 126(20):4553–62. 10.1002/cncr.33050 32770762

[3] Siegel RL Fedewa SA Anderson WF Miller KD Ma J Rosenberg PS. Colorectal Cancer Incidence Patterns in the United States, 1974-2013. J Natl Cancer Inst (2017) 109(8):djw322. 10.1093/jnci/djw322 PMC6059239 28376186

[4] Sung H Siegel RL Rosenberg PS Jemal A. Emerging Cancer Trends Among Young Adults in the USA: Analysis of a Population-Based Cancer Registry. Lancet Public Health (2019) 4(3):e137–47. 10.1016/S2468-2667(18)30267-6 30733056

[5] Yu M Zhan X Yang Z Huang Y. Measuring the Global, Regional, and National Burden of Type 2 Diabetes and the Attributable Risk Factors in All 194 Countries. J Diabetes (2021) 13(8):613–39. 10.1111/1753-0407.13159 33486878

[6] Dankner R Boffetta P Balicer RD Boker LK Sadeh M Berlin A. Time-Dependent Risk of Cancer After a Diabetes Diagnosis in a Cohort of 2.3 Million Adults. Am J Epidemiol (2016) 183(12):1098–106. 10.1093/aje/kwv290 27257115

[7] Pan XF He M Yu C Lv J Guo Y Bian Z. Type 2 Diabetes and Risk of Incident Cancer in China: A Prospective Study Among 0.5 Million Chinese Adults. Am J Epidemiol (2018) 187(7):1380–91. 10.1093/aje/kwx376 PMC6153481 29304221

[8] Reis JP Allen NB Bancks MP Carr JJ Lewis CE Lima JA. Duration of Diabetes and Prediabetes During Adulthood and Subclinical Atherosclerosis and Cardiac Dysfunction in Middle Age: The CARDIA Study. Diabetes Care (2018) 41(4):731–8. 10.2337/dc17-2233 PMC5860835 29317451

[9] Boursi B Finkelman B Giantonio BJ Haynes K Rustgi AK Rhim AD. A Clinical Prediction Model to Assess Risk for Pancreatic Cancer Among Patients With Prediabetes. Eur J Gastroenterol Hepatol (2021). 10.1097/MEG.0000000000002052 PMC8286263 33470698

[10] Onitilo AA Stankowski RV Berg RL Engel JM Glurich I Williams GM. Breast Cancer Incidence Before and After Diagnosis of Type 2 Diabetes Mellitus in Women: Increased Risk in the Prediabetes Phase. Eur J Cancer Prev (2014) 23(2):76–83. 10.1097/CEJ.0b013e32836162aa 23571511

[11] Goto A Yamaji T Sawada N Momozawa Y Kamatani Y Kubo M. Diabetes and Cancer Risk: A Mendelian Randomization Study. Int J Cancer (2020) 146(3):712–9. 10.1002/ijc.32310 PMC6916579 30927373

[12] Zheng J Rutegård M Santoni G Wallner B Johansson I Sund M. Prediabetes and Diabetes in Relation to Risk of Gastric Adenocarcinoma. Br J Cancer (2019) 120(12):1147–52. 10.1038/s41416-019-0470-1 PMC6738058 31061455

[13] Falk RS Tretli S Paulsen JE Sandvik L Erikssen J Heir T. Response to Intravenous Glucose-Tolerance Test and Risk of Cancer: A Long-Term Prospective Cohort Study. EBioMedicine (2017) 21:117–22. 10.1016/j.ebiom.2017.06.018 PMC5514427 28687499

[14] Pradhan AD Manson JE Rifai N Buring JE Ridker PM. C-Reactive Protein, Interleukin 6, and Risk of Developing Type 2 Diabetes Mellitus. JAMA (2001) 286(3):327–34. 10.1001/jama.286.3.327 11466099

[15] Xia B He Q Pan Y Gao F Liu A Tang Y. Metabolic Syndrome and Risk of Pancreatic Cancer: A Population-Based Prospective Cohort Study. Int J Cancer (2020) 147(12):3384–93. 10.1002/ijc.33172 32580250

[16] Park YM Bookwalter DB O’Brien KM Jackson CL Weinberg CR Sandler DP. A Prospective Study of Type 2 Diabetes, Metformin Use, and Risk of Breast Cancer. Ann Oncol (2021) 32(3):351–9. 10.1016/j.annonc.2020.12.008 PMC7995619 33516778

[17] Di Matteo S Nevi L Overi D Landolina N Faccioli J Giulitti F. Metformin Exerts Anti-Cancerogenic Effects and Reverses Epithelial-to-Mesenchymal Transition Trait in Primary Human Intrahepatic Cholangiocarcinoma Cells. Sci Rep (2021) 11(1):2557. 10.1038/s41598-021-81172-0 33510179 PMC7844056

[18] Kurelac I Umesh Ganesh N Iorio M Porcelli AM Gasparre G. The Multifaceted Effects of Metformin on Tumor Microenvironment. Semin Cell Dev Biol (2020) 98:90–7. 10.1016/j.semcdb.2019.05.010 31091466

[19] Shin CM Kim N Han K Kim B Jung JH Oh TJ. Anti-Diabetic Medications and the Risk for Colorectal Cancer: A Population-Based Nested Case-Control Study. Cancer Epidemiol (2020) 64:101658. 10.1016/j.canep.2019.101658 31887708

[20] Xiao L Yin X Di X Nan Y Lyu T Wu Y. Awareness and Prevalence of E-Cigarette Use Among Chinese Adults: Policy Implications. Tob Control (2021). 10.1136/tobaccocontrol-2020-056114 PMC9234419 33608465

[21] Murphy CC Gerber DE Pruitt SL. Prevalence of Prior Cancer Among Persons Newly Diagnosed With Cancer: An Initial Report From the Surveillance, Epidemiology, and End Results Program. JAMA Oncol (2018) 4(6):832–6. 10.1001/jamaoncol.2017.3605 PMC6370034 29167866

[22] Alberti KG Zimmet PZ. Definition, Diagnosis and Classification of Diabetes Mellitus and its Complications. Part 1: Diagnosis and Classification of Diabetes Mellitus Provisional Report of a WHO Consultation. Diabetes Med (1998) 15(7):539–53. 10.1002/(SICI)1096-9136(199807)15:7<539::AID-DIA668>3.0.CO;2-S 9686693

[23] Li X Deng Y Tang W Sun Q Chen Y Yang C. Urban-Rural Disparity in Cancer Incidence, Mortality, and Survivals in Shanghai, China, During 2002 and 2015. Front Oncol (2018) 8:579. 10.3389/fonc.2018.00579 30560091 PMC6287035

[24] Vu HT Ufere N Yan Y Wang JS Early DS Elwing JE. Diabetes Mellitus Increases Risk for Colorectal Adenomas in Younger Patients. World J Gastroenterol (2014) 20(22):6946–52. 10.3748/wjg.v20.i22.6946 PMC4051936 24944487

[25] Restifo D Williams JS Garacci E Walker RJ Ozieh MN Egede LE. Differential Relationship Between Colorectal Cancer and Diabetes in a Nationally Representative Sample of Adults. J Diabetes Complications (2018) 32(9):819–23. 10.1016/j.jdiacomp.2018.06.007 PMC8011301 30099983

[26] Lu S Wang A Miao S Zhang X Jing S Shan T. Association Between Type 2 Diabetes and Cancer Incidence in China: Data in Hospitalized Patients From 2006 to 2013. Ann Transl Med (2020) 8(5):176. 10.21037/atm.2020.01.101 32309323 PMC7154402

[27] Yang WS Chen PC Lin HJ Su TC Hsu HC Chen MF. Association Between Type 2 Diabetes and Cancer Incidence in Taiwan: Data From a Prospective Community-Based Cohort Study. Acta Diabetol (2017) 54(5):455–61. 10.1007/s00592-017-0966-1 28190111

[28] Wang Z Wu Y Wu J Wang M Wang X Wang J. Trends in Prevalence and Incidence of Type 2 Diabetes Among Adults in Beijing, China, From 2008 to 2017. Diabetes Med (2021) 38(9):e14487. 10.1111/dme.14487 33278034

[29] Wang L Gao P Zhang M Huang Z Zhang D Deng Q. Prevalence and Ethnic Pattern of Diabetes and Prediabetes in China in 2013. JAMA (2017) 317(24):2515–23. 10.1001/jama.2017.7596 PMC5815077 28655017

[30] Valent F. Diabetes Mellitus and Cancer of the Digestive Organs: An Italian Population-Based Cohort Study. J Diabetes Complications (2015) 29(8):1056–61. 10.1016/j.jdiacomp.2015.07.017 26275864

[31] Pang Y Kartsonaki C Guo Y Chen Y Yang L Bian Z. Diabetes, Plasma Glucose and Incidence of Colorectal Cancer in Chinese Adults: A Prospective Study of 0.5 Million People. J Epidemiol Community Health (2018) 72(10):919–25. 10.1136/jech-2018-210651 PMC6161653 29970599

[32] Bai B Chen H. Metformin: A Novel Weapon Against Inflammation. Front Pharmacol (2021) 12:622262. 10.3389/fphar.2021.622262 33584319 PMC7880161

[33] Van Alsten SC Rabkin CS Sawada N Shimazu T Charvat H Yamaji T. Metabolic Syndrome, Physical Activity, and Inflammation: A Cross-Sectional Analysis of 110 Circulating Biomarkers in Japanese Adults. Cancer Epidemiol Biomarkers Prev (2020) 29(8):1639–46. 10.1158/1055-9965.EPI-19-1513 PMC7528457 32467351

[34] Yang X Tao S Peng J Zhao J Li S Wu N. High-Sensitivity C-Reactive Protein and Risk of Type 2 Diabetes: A Nationwide Cohort Study and Updated Meta-Analysis. Diabetes Metab Res Rev (2021). 10.1002/dmrr.3446 33686799

[35] Xu CX Zhu HH Zhu YM. Diabetes and Cancer: Associations, Mechanisms, and Implications for Medical Practice. World J Diabetes (2014) 5(3):372–80. 10.4239/wjd.v5.i3.372 PMC4058741 24936258

[36] Ryu TY Park J Scherer PE. Hyperglycemia as a Risk Factor for Cancer Progression. Diabetes Metab J (2014) 38(5):330–6. 10.4093/dmj.2014.38.5.330 PMC4209346 25349819

[37] Benhammou JN Lin J Hussain SK El-Kabany M. Emerging Risk Factors for Nonalcoholic Fatty Liver Disease Associated Hepatocellular Carcinoma. Hepatoma Res (2020) 6:35. 10.20517/2394-5079.2020.16 32685690 PMC7367098

[38] Shlomai G Neel B LeRoith D Gallagher EJ. Type 2 Diabetes Mellitus and Cancer: The Role of Pharmacotherapy. J Clin Oncol (2016) 34(35):4261–9. 10.1200/JCO.2016.67.4044 PMC5455318 27903154

