# Supplementary figures and images for: Glucose Intolerance and Cancer Risk: A Community-Based Prospective Cohort Study in Shanghai, China
Source: Front Oncol. 2021 Aug 30;11:726672. doi: 10.3389/fonc.2021.726672 (PMC8435720; doi:10.3389/fonc.2021.726672)

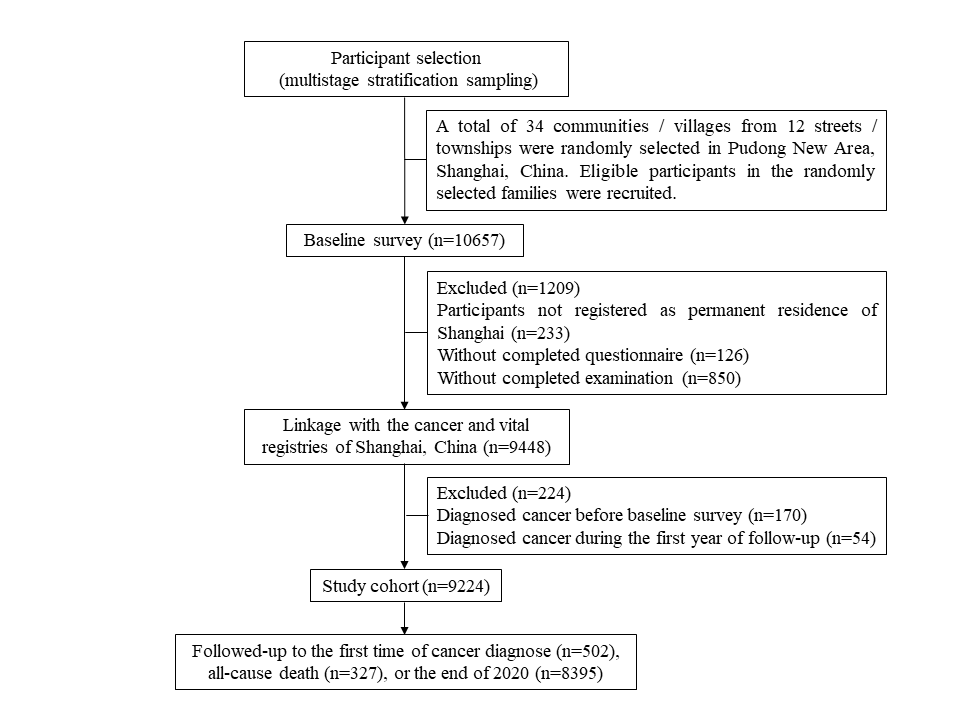

Supplement: Supplementary Figure 1 — Diagram of this community-based prospective cohort study [file Image_1.tif]

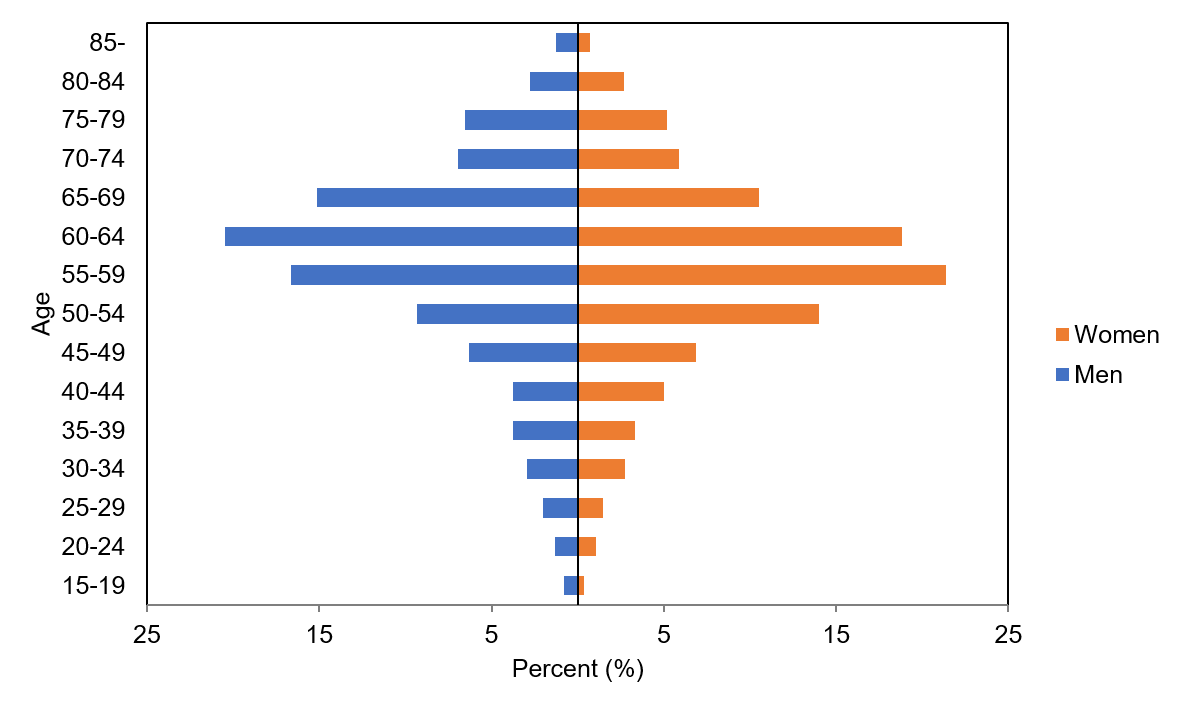

Supplement: Supplementary Figure 2 — Age and sex distribution of study subjects. [file Image_2.tif]
