# Supplementary material for: Glucose Intolerance and Cancer Risk: A Community-Based Prospective Cohort Study in Shanghai, China
Source: Front Oncol. 2021 Aug 30;11:726672. doi: 10.3389/fonc.2021.726672 (PMC8435720; doi:10.3389/fonc.2021.726672)
Supplement: Supplementary file 3 [file Table_1.docx]

**Questionnaire**

According to Article 15, Chapter 3 of the Statistic Law of the People's Republic of China, the information of individual and the family is not allowed to release unless approved by the individual and the legal guardian.

| Name: Contact phone number: | |
| --- | --- |
| Township/Street: _____________ | Serial number of rural township or urban street: _________ |
|  |  |
| village/residents' committee: __________ | Serial number of villages’ or residents' committee: _________ |
| Serial number of family: _____________ | |
| Investigator:  Date of investigation: | Quality controller:  Date of quality control: |

Pudong New Area Center for Disease Control and Prevention

Formulated in 2012

**Family Questionnaire**

**Family members**

Please fill the information of all your family members who participated in this investigation

| Name | Relationship with respondents | sex | age | Personal serial number | Household registered permanent residence:  1.Shanghai  2.Other provinces |
| --- | --- | --- | --- | --- | --- |
|  |  |  |  |  |  |
|  |  |  |  |  |  |
|  |  |  |  |  |  |
|  |  |  |  |  |  |

**Dietary status of your family**

| Q1 | Number of people have breakfast at home |  |
| --- | --- | --- |
| Q2 | Number of people have lunch at home |  |
| Q3 | Number of people have dinner at home |  |
| Q4 | Total quantity of animal oil consumed per month |  |
| Q5 | Total quantity of vegetable oil consumed per month |  |
| Q6 | Total quantity of salt consumed per month |  |
| Q7 | Total quantity of soy sauce consumed per month |  |
| Q8 | Total quantity of sugar consumed per month |  |

**Economic status of your family**

| Q9 | Total family income in 2012 | 1 yuan per month  2 yuan per year  99 Don't know the exact income  97 Refuse to answer |
| --- | --- | --- |
| Q10 | Total family expenditure in 2012 | 1 yuan per month  2 yuan per year  99 Don't know the exact expenditure  97 Refuse to answer |

**Personal Questionnaire**

| Name: Contact phone: | |
| --- | --- |
| Serial number of family: _____________; Identity card number: _____________ | |
| Original personal serial number: _____________ | Current personal serial number: _____________ |
| Start time of investigation: _____________ | |
| Investigator:  Date of investigation: | Quality controller:  Date of quality control: |

Pudong New Area Center for Disease Control and Prevention

Formulated in 2012

**Part 1: Demographic information**

A1. Sex:

1. Male 2. Female

A2. Nationality:

1. Han 88. other

A3. Date of birth:

A4. Years of education:

A5. Marriage status:

A6. Blood group (type):

1. A 2. B 3. AB 4. O 99. Unknown

A7. Occupation:

**Part 2: medical history and family history**

| **Item** | **Disease** | **Yourself diagnosed with this disease (A1-A34)**  **1 Yes 2 No** | **Time of first diagnosis**  **(B1-B34)** | **Your relatives have diagnosed with this disease (C1-C34)**  **1 Yes 2 No** | **Type and number of your relatives diagnosed with this disease** | | | | | | | | | | | | |
| --- | --- | --- | --- | --- | --- | --- | --- | --- | --- | --- | --- | --- | --- | --- | --- | --- | --- |
|  |  |  |  |  | **daughter** | | **son** | | **brother** | | | | **sister** | **father** | | **mother** | |
| Infectious disease | **B1** viral hepatitis |  |  |  |  | |  | |  | | | |  |  | |  | |
|  | **B1** type:1. hepatitis A 2. hepatitis B 3. Hepatitis C 4. Hepatitis D 5. Hepatitis E 99. unknown | | | | | | | | | | | | | | | | |
|  | **B2** tuberculosis |  |  |  |  | |  | | | | |  |  | |  | |  |
| cardiovascular diseases | **B3** hypertension |  |  |  |  | |  | | | | |  |  | |  | |  |
|  | **B4** coronary heart disease |  |  |  |  | |  | | | | |  |  | |  | |  |
|  | **B4B1** type: 1.Angina 2. Myocardial infarction 3. Other 99. Unknown | | | | | | | | | | | | | | | | |
|  | **B5** stroke |  |  |  |  | |  | | | |  | |  |  | |  | |
|  | **B5B1** typer:1. Cerebral hemorrhage 2. Cerebral infarction 3. other 99. unknown | | | | | | | | | | | | | | | | |
|  | **B6** Acute myocardial infarction |  |  |  |  | |  | | | |  | |  |  | |  | |
| Endocrine, nutritional and metabolic diseases | **B7** type 2 diabetes |  |  |  |  | |  | | | |  | |  |  | |  | |
|  | **B8** hyperthyroidism |  |  |  |  | |  | | | |  | |  |  | |  | |
|  | **B9** hypothyroidism |  |  |  |  | |  | | | |  | |  |  | |  | |
|  | **B10** hyperuricemia |  |  |  |  | |  | | | |  | |  |  | |  | |
|  | **B11** dyslipidemia |  |  |  |  | |  | | | |  | |  |  | |  | |
|  | **B11B1** type:1. hypercholesterolemia 2. hypertriglyceridemia 3. mixed type 99. unknown | | | | | | | | | | | | | | | | |
|  | **B12** polycystic ovarian syndrome (women) |  |  |  |  | |  |  | | | | |  |  | |  | |
| Nervous system disease | **B13** Parkinson’s disease |  |  |  |  | |  |  | | | | |  |  | |  | |
| Oculopathy | **B14** Glaucoma |  |  |  |  | |  |  | | | | |  |  | |  | |
|  | **B15** Cataract |  |  |  |  | |  |  | | | | |  |  | |  | |
|  | **B16** Diabetic retinopathy |  |  |  |  | |  |  | | | | |  |  | |  | |
| Respiratory disease | **B17** Chronic bronchitis |  |  |  |  | |  |  | | | | |  |  | |  | |
|  | **B18** Asthma |  |  |  |  | |  |  | | | | |  |  | |  | |
|  | **B19** Chronic obstructive pulmonary disease |  |  |  |  | |  |  | | | | |  |  | |  | |
| Digestive diseases | **B20** Gastric ulcer |  |  |  |  | |  |  | | | | |  |  | |  | |
|  | **B21** Duodenal ulcer |  |  |  |  | |  |  | | | | |  |  | |  | |
|  | **B22** Liver cirrhosis |  |  |  |  | |  |  | | | | |  |  | |  | |
|  | **B23** Fatty liver disease |  |  |  |  | |  |  | | | | |  |  | |  | |
|  | **B24** Chronic atrophic gastritis |  |  |  |  | |  |  | | | | |  |  | |  | |
|  | **B25** Intestinal polyps |  |  |  |  | |  |  | | | | |  |  | |  | |
|  | **B26** Cholecystitis |  |  |  |  | |  |  | | | | |  |  | |  | |
| Urinary diseases | **B27** Chronic renal disease |  |  |  |  | |  |  | | | | |  |  | |  | |
|  | **B27B1** type:1. Nephritis 2. Diabetic nephropathy 3. Hypertensive renal damage 4. Other 99.Unknown | | | | | | | | | | | | | | | | |
| Cancer | **B28** 1: _____ |  |  |  |  |  | | | |  | | |  |  | |  | |
|  | **B29** 2: _____ |  |  |  |  |  | | | |  | | |  |  | |  | |
|  | **B30** 3: _____ |  |  |  |  |  | | | |  | | |  |  | |  | |
| Other diseases | **B31** Allergy |  |  |  |  |  | | | |  | | |  |  | |  | |
|  | **B32** Other1: _____ |  |  |  |  |  | | | |  | | |  |  | |  | |
|  | **B33** Other 2: _____ |  |  |  |  |  | | | |  | | |  |  | |  | |
|  | **B34** Other 3: _____ |  |  |  |  |  | | | |  | | |  |  | |  | |

**Part 3: lifestyle factors**

| C1. Have you ever smoked at least one cigarette a day for more than 6 months? | | | |
| --- | --- | --- | --- |
| 1. Yes→ | C2. when did you started smoking at least one cigarette a day? | | |
|  | C3. Usually how many cigarettes do you smoke per day? | | |
|  | C4. Do you often smoke now? | | |
| 2. No | 2. No→ C5. when did you stopped smoking? (jump to C9) | | |
| (jump to C9) | 1. Yes→ | C6. In the past year, have you ever tried to quit smoking? | |
|  |  | 1. Yes→ | C7. How many times have you tried? |
|  |  | 2. No  (jump to C9) | C8. Longest duration of quit smoking _____ days. 1  or weeks. 2  or months. 3 |

| C9. How old were you when you were exposed to passive smoking? ___________ (if none, jump to C21) C10. Before the age of 20 years, did anyone in your family smoke around you? 1. Yes→  2. No  (jump to C13)  C13. After the age of 20, did anyone in your family smoke around you？  1. Yes→  2. No  (jump to C17) |
| --- |

| C17. Were you exposed to passive smoking at your workplace?  1. Yes→  2. No  (jump to C21) |
| --- |

| C21. Have you been drinking alcohol at least 3 times a week for at least 6 months？ | | | |  |
| --- | --- | --- | --- | --- |
| 1. Yes→ | C22. When did you started drinking? | | |  |
|  | C23. Did you drink alcohol regularly in the past 12 months? | | |  |
|  | 1. Yes→ | | C24. How many times per week did you drink alcohol in the past 12 months? |  |
|  | 2. No | |  |  |
|  | (jump to C26) | | C25. How many did you drink per week in the past 12 months? |  |
| 2. No  (jump to C27) |  | | 1. yellow wine or rice wine  2. beer  3. liquor  4. wine |  |
|  | C26. when did you stopped drinking? | | |  |
| C27. Have you been drinking tea at least 3 times a week for at least 6 months？ | | | | |
| 1. Yes→ | | C28. When did you started drinking? | | |
|  | | C29. Did you drink tea regularly in the past 12 months? | | |
| 2. No  (jump to C33) | | 1. Yes→  2. No  (jump to C31) | C30. Which kind of tea did you usually drink?  1. green tea  2. black tea  3. dark tea  88. other | |
|  | | C31. when did you stopped drinking tea?  C32. How much tea did you drink per week in the past 12 months? | | |

**Part 4: physical activities**

| D1. In the past 5 years, have you often participated in sports activities? | | | 1. Yes  2. No (jump to D11) | |  |
| --- | --- | --- | --- | --- | --- |
| D2. Do you engage in vigorous physical activity at least once a week? | | | 1. Yes  2. No (jump to D6) | |  |
| D3. Type of vigorous physical activities: (D3A1. D3A3)  1:  2:  3: | D4. Duration of physical activities: (D4B1. D4B3)  hours  hours  hours | | D5. Doing this for how many years: (D5C1. D5C3)  years  years  years |  |  |
|  | | | |  |  |
| D6. Do you engage in moderate physical activity at least once a week? | | | 1. Yes  2. No (jump to D10) |  |  |
| D7. Type of moderate physical activities: (D7A1. D7A3)  1:  2:  3: | | D8. Duration of physical activities: (D8B1. D8B3)  hours  hours  hours | D9. Doing this for how many years: (D9C1. D9C3)  years  years  years | | |

**Part 5: medications**

| **Medication** | **A. Have you ever taken the medication**  **1 Yes 2 No** | **B. How long did you take the medication(months)** |
| --- | --- | --- |
| **Antihypertensive agents** | | |
| E1. diuretic |  |  |
| E2. angiotensin-converting-enzyme inhibitors |  |  |
| E3. angiotensin receptor inhibitors |  |  |
| E4. β-receptor inhibitors |  |  |
| E5. calcium channel blocker |  |  |
| E6. α-receptor inhibitors |  |  |
| E7. other antihypertensive agents |  |  |
| **Antidiabetic agents** | | |
| E8. insulin |  |  |
| E9. euglycemic agents |  |  |
| E10. sulfonylureas |  |  |
| E11. non-sulfonylureas |  |  |
| E12. biguanides |  |  |
| E13. thiazolidinediones |  |  |
| E14. α-glycosidase inhibitors |  |  |
| E15. traditional Chinese medicine |  |  |
| E16. other antidiabetic agents |  |  |
| **Hypolipidemic agents** | | |
| E17. phenoxy aromatics |  |  |
| E18. statins |  |  |
| E19. nicotinic acid niacin |  |  |
| E20. polyunsaturated fatty acids |  |  |
| E21. pantetheines |  |  |
| E22. polysaccharide sulfate |  |  |
| E23. traditional Chinese medicine |  |  |
| **Nonsteroidal antipyretic painkillers** | | |
| E24. aspirin, diclofenac |  |  |
| E25. Other nonsteroidal antipyretic painkillers |  |  |
| **Other medications** | | |
| E31. 1: |  |  |
| E32. 2: |  |  |
| E33. 3: |  |  |

**Part 6: body measurements**

| **Item** | **H1.** **First reading**  **H1A1-H1A13** | **H2.** **Second reading**  **H2B1-H2B13** | **maximum permissible errors** | **H3.** **Third reading**  **H3C1-H3C13** | **Remarks** |
| --- | --- | --- | --- | --- | --- |
| 1.Height(cm) |  |  | 1cm |  |  |
| 2. Weight(kg) |  |  | 1kg |  |  |
| 3. Waist circumference (cm) |  |  | 1cm |  |  |
| 4. Hip circumference (cm) |  |  | 1cm |  |  |
| 5. Systolic pressure of the right arm (mmHg) |  |  | 1mmHg |  |  |
| 6. Diastolic pressure of the right arm (mmHg) |  |  | 1mmHg |  |  |
| 7. Systolic pressure of the left arm (mmHg) |  |  | 1mmHg |  |  |
| 8. Diastolic pressure of the left arm (mmHg) |  |  | 1mmHg |  |  |
| 9. Heart rate (times per minute) |  |  | 1time |  |  |

If the first two readings exceed the maximum permissible error, take the third measure and record the third reading.

**Postscript of investigator**

I1. Respondents' willingness to participate in the follow-up interview:

1. Yes 2. No 3. Not sure

I2. For the convenience of contact, please tell us an address of your relatives or friends

name: Relationship to you: _______ phone number:

address:

I3. Respondents' cooperative attitude:

1. very good 2. good 3. general 4. poor

I4. Credibility of the questionnaire:

1. very good 2. good 3. general 4. poor

I5. Location of investigation:

1. hospital 2. workplace 3. Respondents' home 4. other

I6. The relationship between the investigation subject and the survey subject:

1. self 2. spouse 3. parent 4. offspring 5. brothers or sisters 88. other

I7. Time of completion of the questionnaire:
