# Supplementary material for: Glucose Intolerance and Cancer Risk: A Community-Based Prospective Cohort Study in Shanghai, China
Source: Front Oncol. 2021 Aug 30;11:726672. doi: 10.3389/fonc.2021.726672 (PMC8435720; doi:10.3389/fonc.2021.726672)
Supplement: Supplementary file 4 [file Table_2.docx]

Supplementary Table 2. The Cox regression analysis of the association of site-specific cancer with the baseline glycemic status in the whole cohort, adjusted for age and sex

| Glycemic status | Persons at risk | Incident cases | Person-years | Incidence  (1/ 1000) | HR (95% CI) | *p* |
| --- | --- | --- | --- | --- | --- | --- |
| Lung cancer | | | | | | |
| NGT | 5980 | 68 | 45184 | 1.50 | ref. |  |
| Glucose intolerance | 3244 | 46 | 23783 | 1.93 | 1.02(0.70-1.50) | 0.916 |
| Female breast cancer | | | | | | |
| NGT | 3873 | 27 | 29370 | 0.92 | ref. |  |
| Glucose intolerance | 1956 | 26 | 14449 | 1.80 | 1.95(1.14-3.34) | 0.015 |
| Stomach cancer | | | | | | |
| NGT | 5980 | 20 | 45184 | 0.44 | ref. |  |
| Glucose intolerance | 3244 | 24 | 23783 | 1.01 | 1.78(0.97-3.26) | 0.063 |
| Colorectal cancer | | | | | | |
| NGT | 5980 | 23 | 45184 | 0.51 | ref. |  |
| Glucose intolerance | 3244 | 24 | 23783 | 1.01 | 1.39(0.78-2.49) | 0.266 |
| Kidney cancer | | | | | | |
| NGT | 5980 | 2 | 45184 | 0.04 | ref. |  |
| Glucose intolerance | 3244 | 10 | 23783 | 0.42 | 8.63(1.89-39.41) | 0.005 |
| Liver cancer | | | | | | |
| NGT | 5980 | 13 | 45184 | 0.29 | ref. |  |
| Glucose intolerance | 3244 | 9 | 23783 | 0.38 | 0.84(0.36-2.00) | 0.700 |
| Pancreatic cancer | | | | | | |
| NGT | 5980 | 6 | 45184 | 0.13 | ref. |  |
| Glucose intolerance | 3244 | 8 | 23783 | 0.34 | 2.52(0.87-7.26) | 0.087 |
| Esophageal cancer | | | | | | |
| NGT | 5980 | 8 | 45184 | 0.18 | ref. |  |
| Glucose intolerance | 3244 | 3 | 23783 | 0.13 | 0.44(0.11-1.67) | 0.226 |

HR, Hazard ratio; NGT, normal glucose tolerance; Glucose intolerance, prediabetes + diabetes.
