# Supplementary material for: Glucose Intolerance and Cancer Risk: A Community-Based Prospective Cohort Study in Shanghai, China
Source: Front Oncol. 2021 Aug 30;11:726672. doi: 10.3389/fonc.2021.726672 (PMC8435720; doi:10.3389/fonc.2021.726672)
Supplement: Supplementary file 5 [file Table_3.docx]

Supplementary Table 3. The Cox regression analysis of the association of site-specific cancer with the baseline glycemic status in the whole cohort, adjusted for age and sex

| Glycemic status | Persons at risk | Incident cases | Person-years | Incidence (1/ 1000) | HR (95% CI) | *p* |
| --- | --- | --- | --- | --- | --- | --- |
| Lung cancer | | | | | | |
| NGT | 5980 | 68 | 45184 | 1.50 | ref. |  |
| Prediabetes | 1454 | 22 | 10756 | 2.05 | 1.10(0.67-1.79) | 0.707 |
| Diabetes | 1790 | 24 | 13027 | 1.84 | 0.96(0.6-1.54) | 0.861 |
| Female breast cancer | | | | | | |
| NGT | 3873 | 27 | 29370 | 0.92 | ref. |  |
| Prediabetes | 910 | 12 | 6807 | 1.76 | 1.91(0.97-3.78) | 0.062 |
| Diabetes | 1046 | 14 | 7642 | 1.83 | 1.98(1.04-3.78) | 0.038 |
| Stomach cancer | | | | | | |
| NGT | 5980 | 20 | 45184 | 0.44 | ref. |  |
| Prediabetes | 1454 | 13 | 10756 | 1.21 | 2.18(1.07-4.43) | 0.032 |
| Diabetes | 1790 | 11 | 13027 | 0.84 | 1.46(0.69-3.08) | 0.321 |
| Colorectal cancer | | | | | | |
| NGT | 5980 | 23 | 45184 | 0.51 | ref. |  |
| Prediabetes | 1454 | 8 | 10756 | 0.74 | 1.06(0.47-2.38) | 0.896 |
| Diabetes | 1790 | 16 | 13027 | 1.23 | 1.66(0.87-3.17) | 0.127 |
| Kidney cancer | | | | | | |
| NGT | 5980 | 2 | 45184 | 0.04 | ref. |  |
| Prediabetes | 1454 | 5 | 10756 | 0.46 | 10.11(1.96-52.10) | 0.006 |
| Diabetes | 1790 | 5 | 13027 | 0.38 | 7.53(1.46-38.84) | 0.016 |
| Liver cancer | | | | | | |
| NGT | 5980 | 13 | 45184 | 0.29 | ref. |  |
| Prediabetes | 1454 | 2 | 10756 | 0.19 | 0.43(0.10-1.90) | 0.263 |
| Diabetes | 1790 | 7 | 13027 | 0.54 | 1.17(0.46-2.98) | 0.735 |
| Pancreatic cancer | | | | | | |
| NGT | 5980 | 6 | 45184 | 0.13 | ref. |  |
| Prediabetes | 1454 | 3 | 10756 | 0.28 | 2.09(0.52-8.37) | 0.296 |
| Diabetes | 1790 | 5 | 13027 | 0.38 | 2.87(0.88-9.40) | 0.082 |
| Esophagus cancer | | | | | | |
| NGT | 5980 | 8 | 45184 | 0.18 | ref. |  |
| Prediabetes | 1454 | 1 | 10756 | 0.09 | 0.33(0.04-2.69) | 0.302 |
| Diabetes | 1790 | 2 | 13027 | 0.15 | 0.52(0.11-2.46) | 0.407 |

HR, Hazard ratio; NGT, normal glucose tolerance.
