# Supplementary material for: Glucose Intolerance and Cancer Risk: A Community-Based Prospective Cohort Study in Shanghai, China
Source: Front Oncol. 2021 Aug 30;11:726672. doi: 10.3389/fonc.2021.726672 (PMC8435720; doi:10.3389/fonc.2021.726672)
Supplement: Supplementary file 6 [file Table_4.docx]

Supplementary Table 4. Hazard ratios for site-specific cancer incidence according to the baseline glycemic status in young adults (aged 15-64 years).

| Glycemic status | Persons at risk | Incident cases | Person-years | Incidence (1/ 1000) | HR (95% CI) | *p* |
| --- | --- | --- | --- | --- | --- | --- |
| Lung cancer | | | | | | |
| NGT | 4701 | 45 | 35880 | 1.25 | ref. |  |
| Prediabetes | 917 | 10 | 6892 | 1.45 | 0.98(0.49-1.96) | 0.961 |
| Diabetes | 1043 | 13 | 7808 | 1.66 | 1.09(0.59-2.04) | 0.777 |
| Female breast cancer | | | | | | |
| NGT | 3163 | 23 | 24123 | 0.95 | ref. |  |
| Prediabetes | 608 | 8 | 4584 | 1.75 | 1.59(0.71-3.57) | 0.263 |
| Diabetes | 603 | 8 | 4523 | 1.77 | 1.53(0.67-3.45) | 0.310 |
| Stomach cancer | | | | | | |
| NGT | 4701 | 10 | 35880 | 0.28 | ref. |  |
| Prediabetes | 917 | 8 | 6892 | 1.16 | 4.14(1.63-10.48) | 0.003 |
| Diabetes | 1043 | 8 | 7808 | 1.02 | 3.37(1.33-8.57) | 0.011 |
| Colorectal cancer | | | | | | |
| NGT | 4701 | 9 | 35880 | 0.25 | ref. |  |
| Prediabetes | 917 | 3 | 6892 | 0.44 | 1.73(0.47-6.39) | 0.411 |
| Diabetes | 1043 | 10 | 7808 | 1.28 | 5.08(2.07-12.51) | 0.000 |
| Kidney cancer | | | | | | |
| NGT | 4701 | 2 | 35880 | 0.06 | ref. |  |
| Prediabetes | 917 | 4 | 6892 | 0.58 | 10.3(1.89-56.27) | 0.007 |
| Diabetes | 1043 | 4 | 7808 | 0.51 | 7.51(1.37-41.05) | 0.020 |
| Liver cancer | | | | | | |
| NGT | 4701 | 5 | 35880 | 0.14 | ref. |  |
| Prediabetes | 917 | 2 | 6892 | 0.29 | 2.09(0.41-10.77) | 0.379 |
| Diabetes | 1043 | 1 | 7808 | 0.13 | 0.92(0.11-7.88) | 0.940 |
| Pancreatic cancer | | | | | | |
| NGT | 4701 | 3 | 35880 | 0.08 | ref. |  |
| Prediabetes | 917 | 3 | 6892 | 0.44 | 5.23(1.06-25.91) | 0.043 |
| Diabetes | 1043 | 1 | 7808 | 0.13 | 1.54(0.16-14.79) | 0.709 |
| Esophageal cancer | | | | | | |
| NGT | 4701 | 3 | 35880 | 0.08 | - | - |
| Prediabetes | 917 | 0 | 6892 | 0.00 | - | - |
| Diabetes | 1043 | 0 | 7808 | 0.00 | - | - |

HR, Hazard ratio; NGT, normal glucose tolerance.
